# Data supporting that adipose-derived mesenchymal stem/stromal cells express angiotensin II receptors in situ and in vitro

**DOI:** 10.1016/j.dib.2017.11.058

**Published:** 2017-11-21

**Authors:** Liudmila V. Ageeva, Veronika Y. Sysoeva, Pyotr A. Tyurin-Kuzmin, George V. Sharonov, Daniyar T. Dyikanov, Dmitry V. Stambolsky, Natalia I. Kalinina

**Affiliations:** Department of Biochemistry and Molecular Medicine, Faculty of Medicine, Lomonosov Moscow State University, 27-1 Lomonosovsky av, Moscow 119192 Russia

## Abstract

This article contains results of analyses of angiotensin II receptors expression in human adipose tissue and stem/stromal cells isolated from adipose tissue. We also provide here data regarding the effect of angiotensin II on intracellular calcium mobilization in adipose tissue derived stem/stromal cells (ADSCs). Discussion of the data can be found in (Sysoeva et al., 2017) [1].

**Specifications Table**

**Table:** 

Subject area	Biology
More specific subject area	Mesenchymal stem/stromal cells; adipose differentiation
Type of data	Confocal and fluorescent microscopy images, flow cytometry data, table
How data was acquired	Images were obtained by confocal microscope LSM 780 and ZEN2010 software (Zeiss). Flow cytometry data were acquired by LSR Fortessa flow cytometer and FACSDiva software (BD). Ca2+ transients were measured in individual cells using fluorescent microscope Eclipse Ti and analyzed using NIS-Elements software (Nikon).
Data format	Analyzed
Experimental factors	ADSC pre-treated with human AngII in the presence or absence of losartan (AngII type 1 receptor inhibitor)
Experimental features	The expression of angiotensin type 1, type 2 and MAS1 receptors was analysed in adipose tissue in situ and in cultured ADSCs. AngII was applied to cultured ADSC and intracellular Ca2+ transients were measured in individual cells
Data source location	Lomonosov Moscow State University, Moscow, Russia
Data accessibility	Data with this article
Related research article	Sysoeva V. et al. Local angiotensin II promotes adipogenic differentiation of human adipose tissue mesenchymal stem cells through type 2 angiotensin receptor. Stem Cell Res. in press

**Value of the data**

• Data regarding the in situ expression of angiotensin II receptors will help to pursue the role of angiotensin II in the adipose tissue growth and renewal
• Angiotensin II triggers intracellular Ca2+ mobilization in about 5% of ADSCs
• Co-expression of angiotensin II receptors labels particular ADSCs subpopulation

## 1 Data

**Fig. 1:**
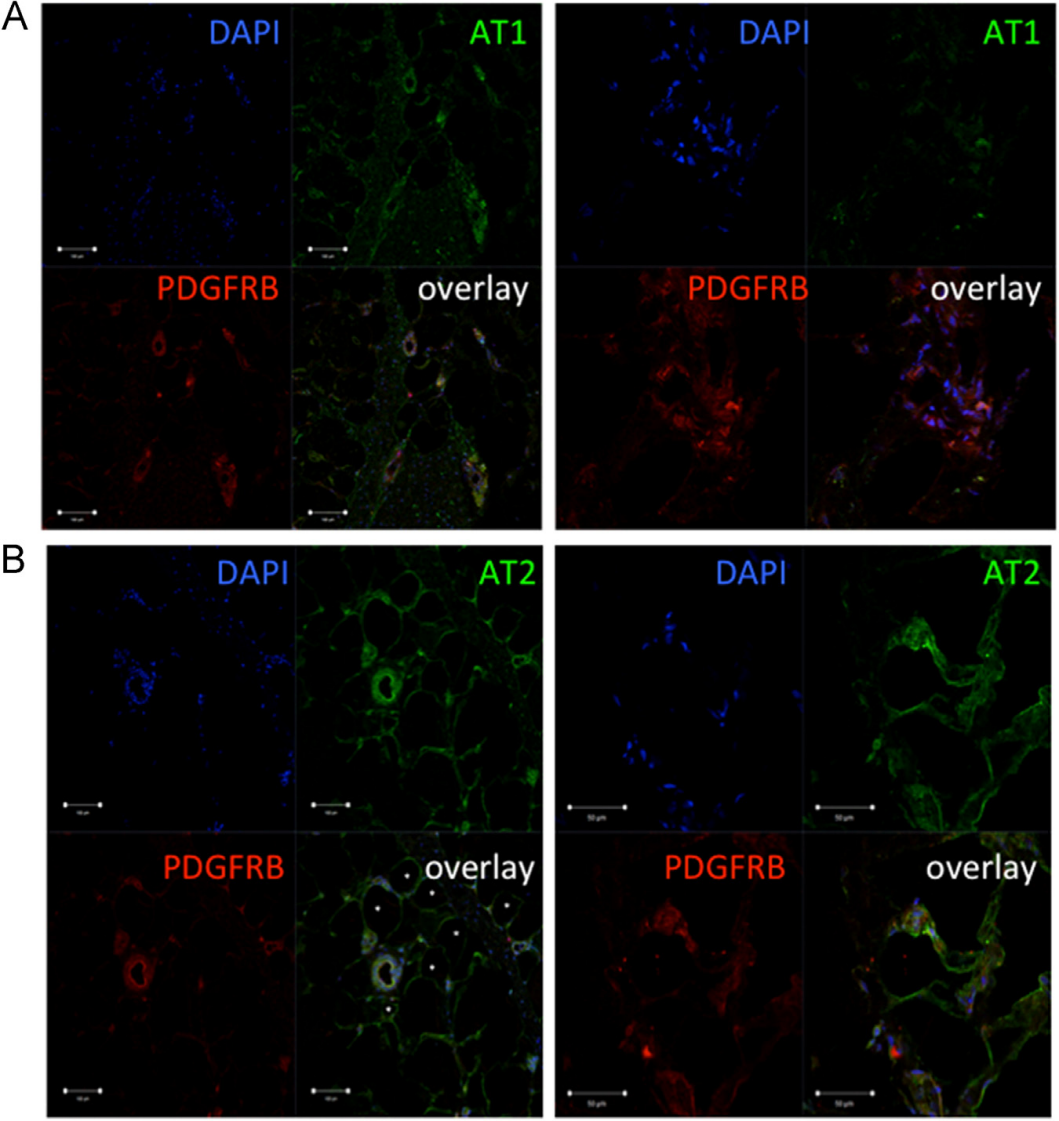
Immunofluorescent staining of frozen adipose tissue sections with antibodies against type 1 (A.) and type 2 (B.) angiotensin receptor (green fluorescence) and ADSCs marker PDGFRbeta (green fluorescence). Scale bars on left panels correspond to 100 µm, on right panels - to 50 µm. Nuclei are counterstained with DAPI (blue fluorescence).

**Fig. 2:**
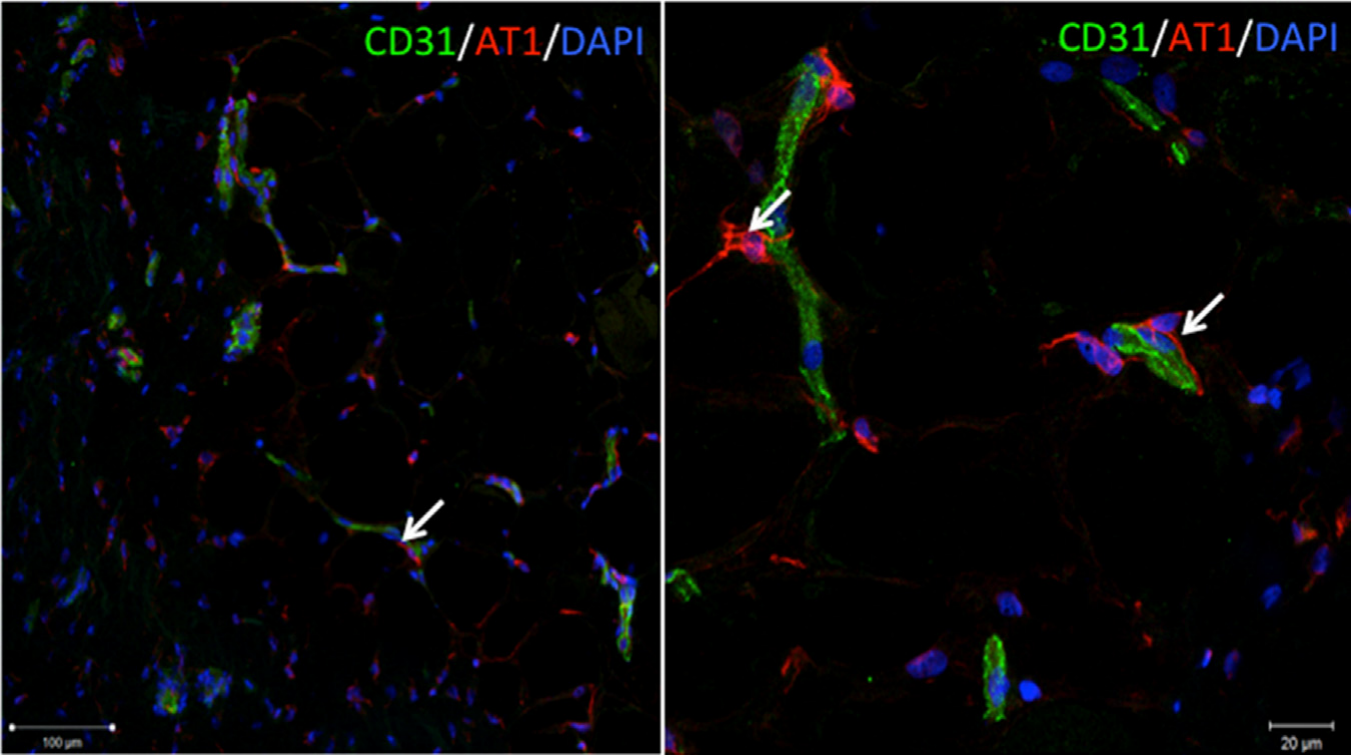
Immunofluorescent staining of frozen adipose tissue sections with antibodies against type 1 angiotensin receptor (red fluorescence) and endothelial cell marker CD31 (green fluorescence). Scale bar on the left panel corresponds to 100 µm, on right panels - to 20 µm. Nuclei are counterstained with DAPI (blue fluorescence).

**Fig. 3:**
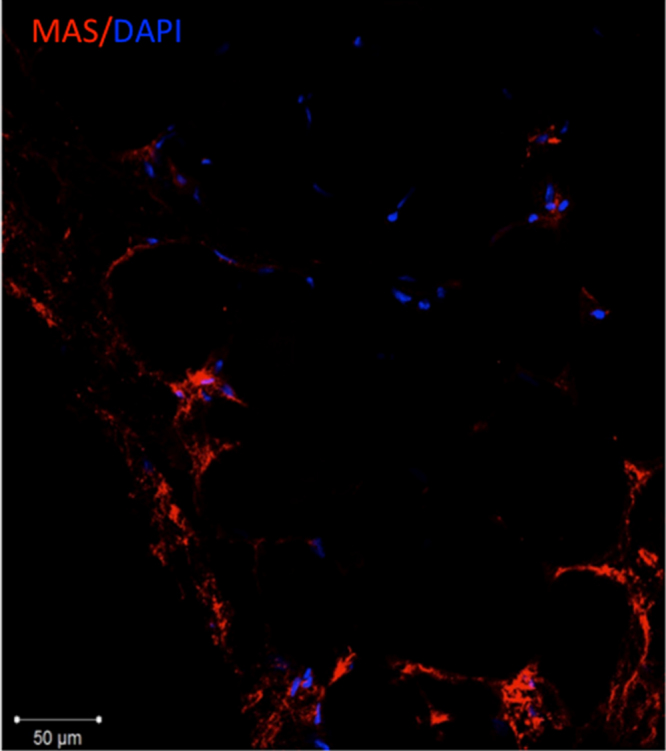
Immunofluorescent staining of frozen adipose tissue sections with antibodies against MAS1 receptor (red fluorescence). The scale bar corresponds to 50 µm. Nuclei are counterstained with DAPI (blue fluorescence).

Using antibodies against AngII receptors (AT1, AT2 and MAS1) we have detected their distribution in human adipose tissue [1]. Specifically, we demonstrated that AT1 and AT2 receptors are co-localized with stromal cells expressing PDGF receptor beta (Fig. 1). Furthermore, AT1 expressing cells were found in the close proximity to CD31-positive endothelial cells (Fig. 2). We also found that adipose tissue contains cells expressing MAS1 receptor (Fig. 3).

**Fig. 4:**
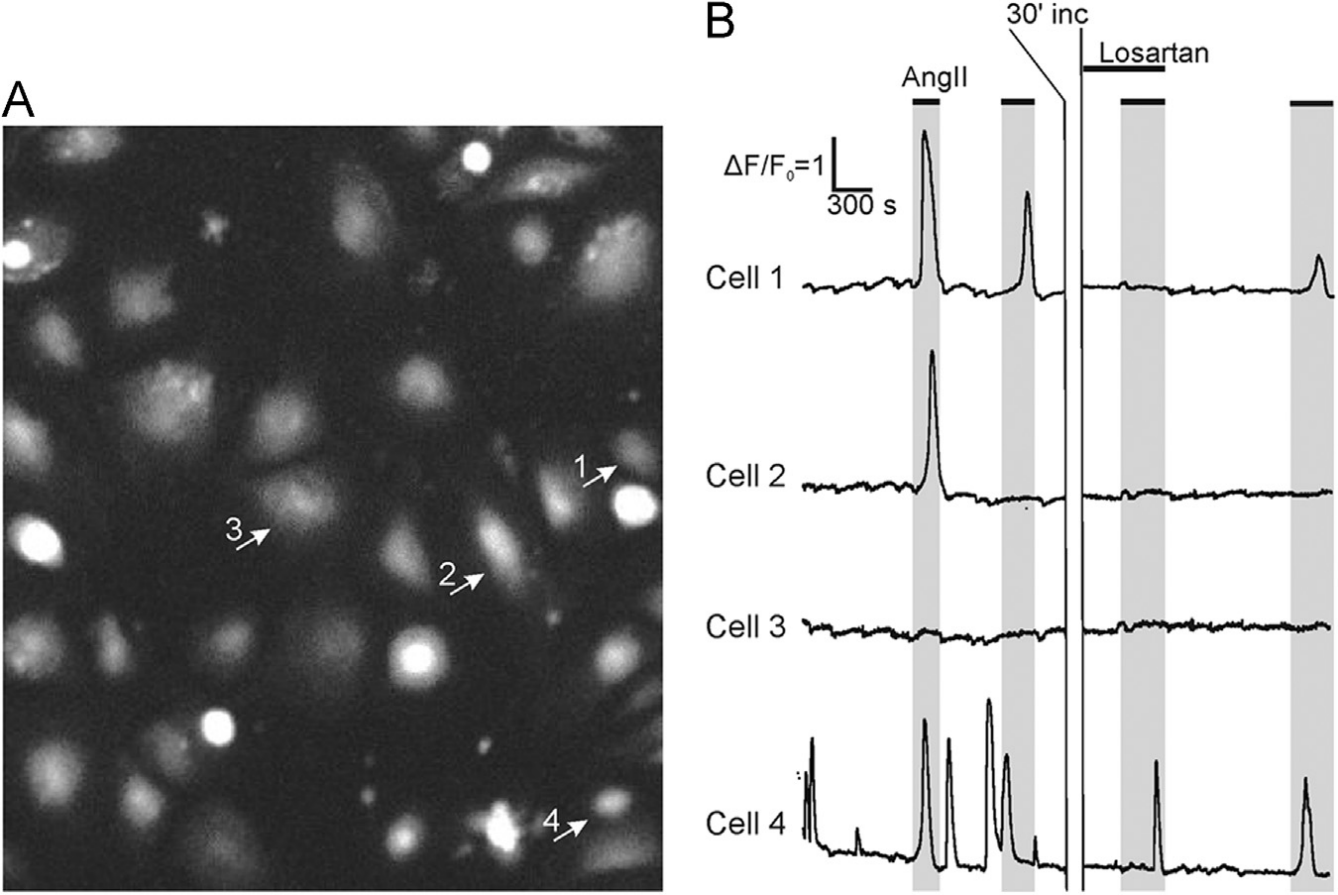
Representative image (A.) and recordings (B.) of intracellular Ca2+ transients in individual ADSCs, treated with Ang II (10 nM) in series in the presence of AT1 receptor antagonist losartan. Short lines above fluorescence trace show applications of indicated compounds. ∆*F*/*F*0 = 1 – fluorescence level of the cell without treatment.

We isolated ADSCs from adipose tissue and examined their ability to respond to AngII. Using fluorescent probe Fluo-8 Ca2+ transients were recorded in ADSCs incubated with AngII in the presence or absence of AT1 inhibitor losartan. We showed that only 5.2 ± 2.7% of cells responded not to single but serial Ang II applications (Fig. 4), whereas in the rest of cells receptors underwent rapid internalization upon the ligand binding [1].

**Fig. 5:**
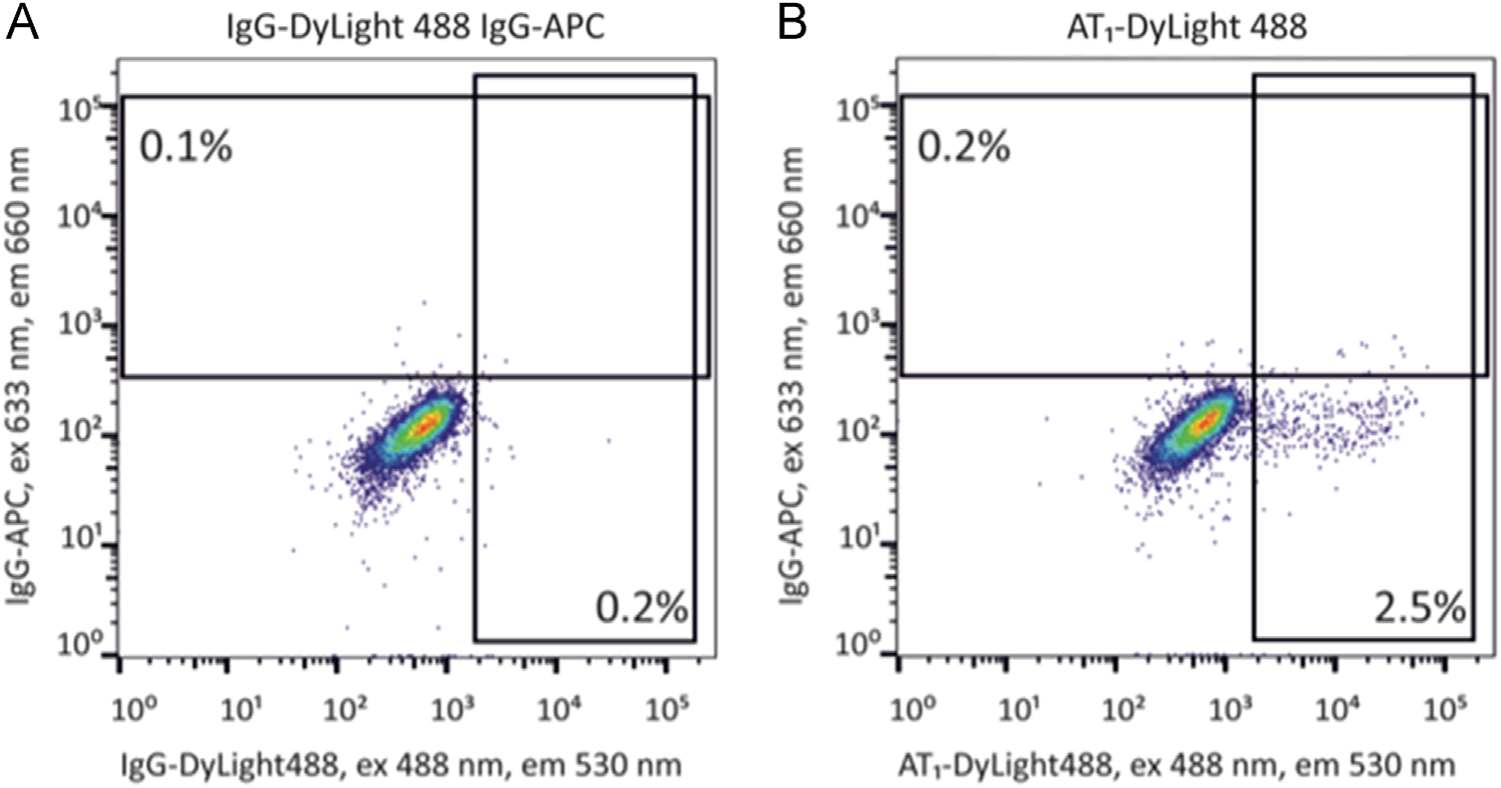
Expression of AT1 receptor on cultured ADSCs. Representative flow cytometry plots. Staining at room temperature. A- IgG-APC, IgG-DyLight-488. B – antibodies against AT1 receptor.

**Fig. 6:**
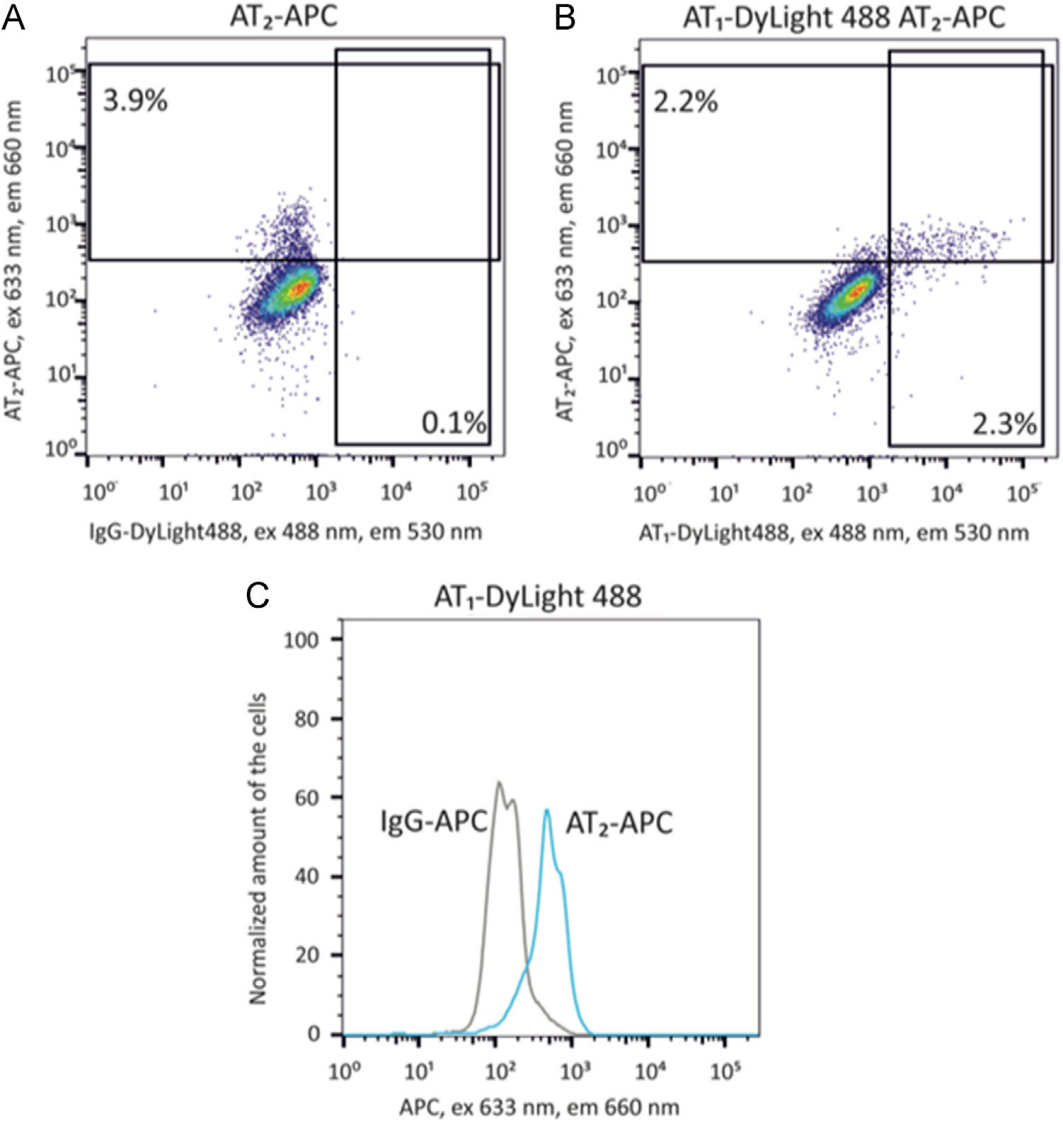
Expression of AT1 receptor on cultured ADSCs. Representative flow cytometry plots. Staining at room temperature. A. – AT2 receptor staining. B. – Double staining of AT1 and AT2 receptors. C. – AT2 expression in ADSCs stably expressing AT1. AT1+-cells are shown only, AT1 receptor individual staining – gray curve, AT1 and AT2 receptor double staining – blue curve. Flow cytometry analysis of ADSCs, stained with antibodies against AT2 receptor.

To evaluate the proportion of ADSCs stably expressing AT1 receptor we provoked its internalization by incubating cells with antibodies against AT1 receptor at the room temperature instead of + 4 °C. we found that in these conditions only 2.8 ± 0.9% of cells were positively stained for AT1 receptor (Fig. 5). We also examined the expression of AT2 receptor and found that proportions of cells expressing AT2 receptors varied substantially between different donors (Table 1). Using flow cytometry we demonstrated that AT2 receptor was predominantly expressed on ADSCs stably expressing AT1 receptor rather than on the other cells in the population (Fig. 6).

**Table 1:** Proportions of ADSCs expressing AT2 receptor in various donors.

**Donor #**	**Proportion of AT2+ cells, %**
3	15,7
4	3
5	2,7
6	4,2
7	9,5
8	1,3
**Average** 5,7	
**SD** 4,7	

## 2 Experimental design, materials, and methods

### 2.1 Immunofluorescent detection of angiotensin II receptors in situ

Subcutaneous fat tissue was obtained from 18 donors during abdominal surgery. All donors gave their informed consent and the local ethics committee of city clinical hospital #31 (Moscow, Russia) approved the study protocol. Part of each sample was placed in O.C.T. Compound (Sakura Inc., Tokyo, Japan) and frozen in liquid nitrogen for immunofluorescent analysis. Other part of each sample was used for ADSC isolation (see below). Angiotensin receptors were visualized by immunofluorescent staining of 10 μm frozen sections using mix of antibodies against AT1 receptor (rabbit polyclonal, PA5-20812, ThermoFisher Scientific) or AT2 receptors (rabbit polyclonal, Alomon) and mouse PDGF receptor beta to stromal cells or CD31 antibodies (BD Pharmingen) to endothelial cells. This was followed by incubation with Alexa594-conjugated donkey anti-rabbit antibody and Alexa488-conjugated anti-mouse antibody (Molecular Probes). Cell nuclei were counterstained with DAPI (Molecular Probes), and sections were mounted in Aqua Poly/Mount (Polysciences Inc). For negative controls mouse or rabbit non-specific IgGs were used in appropriate concentration. Images were obtained using confocal microscope LSM 780 and ZEN2010 software (Zeiss).

### 2.2 ADSCs isolation and culturing

ADSCs were isolated from subcutaneous fat tissue using enzymatic digestion as previously described [2]. Cells were cultured in Mesenchymal Stem Cell Basal Medium (HyClone, GE Healthcare Life Sciences, USA) containing 10% AdvanceSTEM Supplement (HyClone, GE Healthcare Life Sciences, USA), 1% antibiotic–antimycotic solution (HyClone, GE Healthcare Life Sciences, USA) at 37 °C in 5% CO2 incubator. Cells were passaged at 70% confluency using HyQTase Cell Detachment Reagent (HyClone, GE Healthcare Life Sciences, USA). For the experiments, ADSCs cultured up to 2rd–5th passages were used. To confirm their multipotency and specific immunophenotype, ADSCs were induced into osteogenic, adipogenic and chondrogenic differentiation and characterized by the expression of CD45, CD73, CD90, CD105 markers as described earlier [3].

### 2.3 Ca2+ imaging

AT1 receptor activation was assessed using Ca2+ imaging in individual cells as previously described [4]. Briefly, cells were seeded in 24-well plate at low density to prevent cell-to-cell communications during the imaging. ADSCs were loaded with Fluo-8 (ab142773, Abcam), 4 μM in Hanks Balanced Salt Solution with 20 mM HEPES, for 1 h. To stimulate Ca2+ transients in serial mode ADSCs were treated by Ang II (ab120183, Abcam, 10 nM) alone or together with AT1 receptor antagonist losartan (ab120997, Abcam, 1 μM). Losartan was added 30 min before Ang II addition for metabolisation in the cells. Cells were stimulated with Ang II for 4 min, and then washed by Hanks solution 3–5 times for 5 min following by the next stimulation with Ang II. Threshold concentration was determined using addition of 0.1 nM – 10 μM Ang II in series. Ca2+ transients were measured in individual cells using fluorescent microscope Nikon Eclipse Ti (objective 10×) equipped with camera Andor iXon 897 (Andor Technology). Movies were analyzed using NIS-Elements (Nikon) and ImageJ software. Alterations of cytosolic Ca2+ from the resting level were quantified as a difference of Fluo-8 fluorescence intensity (Δ*F*/*F*0) recorded from an individual cell.

### 2.4 Detection of angiotensin II receptors in vitro by flow cytometry

The proportion of ADSCs expressing Ang II receptors was analyzed using flow cytometry. Cells were detached from culture dishes using HyQTase Detachment Reagent (HyClone, GE Healthcare Life Sciences, USA) and stained with antibodies in appropriate combinations. AT1 receptors were detected using specific antibodies AT1 (PA5-20812, ThermoFisher Scientific, dilution 1:100), following by secondary antibodies DyLight 488 (711-485-152, Jackson Immunoresearch, 1:300) and AlexaFluor 594 (111–586-045, Jackson Immunoresearch, 1:500) or AlexaFluor 647 (711-606-152, Jackson Immunoresearch, 1:1000). AT2 receptors were detected by AT2-APC (FAB3659A, R&D Systems, 1:50), following by secondary antibodies DyLight 649 (715-495-150, Jackson Immunoresearch, 1:500). Rabbit IgG Isotype Control (10500C, Invitrogen, 1:170) and APC Mouse IgG2b Control (IC0041A, R&D Systems, 1:50), were used as negative controls. Stained cells were washed once with PBS and analyzed live using a LSR Fortessa flow cytometer (BD Biosciences) and FACSDiva software (BD). The flow cytometry data was analyzed using FlowJo software (FLOWJO, LLC).
