# Supplementary material for: Data supporting that adipose-derived mesenchymal stem/stromal cells express angiotensin II receptors in situ and in vitro
Source: Data Brief. 2017 Nov 21;16:327–33. doi: 10.1016/j.dib.2017.11.058 (PMC5723284; doi:10.1016/j.dib.2017.11.058)
Supplement: Supplementary file 2 — Supplementary material [file mmc2.docx]

Supplementary material


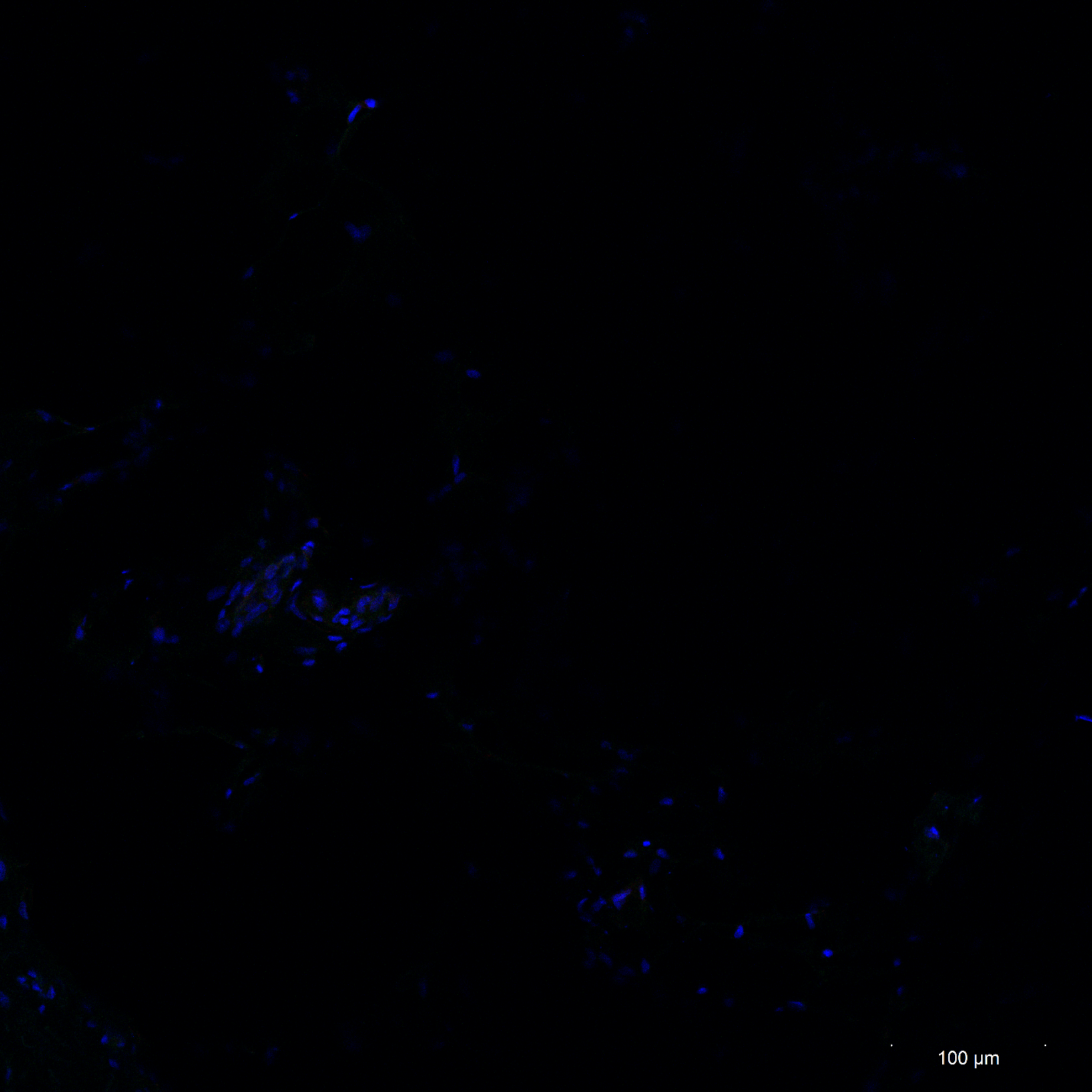


Supplementary figure. Staining of frozen adipose tissue sections with rabbit IgGs . Nuclei are counterstained with DAPI (blue fluorescence).
